# Diagnostic laboratory testing for Charcot Marie Tooth disease (CMT): the spectrum of gene defects in Norwegian patients with CMT and its implications for future genetic test strategies

**DOI:** 10.1186/1471-2350-14-94

**Published:** 2013-09-21

**Authors:** Rune Østern, Toril Fagerheim, Helene Hjellnes, Bjørn Nygård, Svein I Mellgren, Øivind Nilssen

**Affiliations:** 1 Department of Medical Genetics, University Hospital of North-Norway, Tromsø NO9038, Norway; 2 Department of Clinical Medicine, Neuromuscular Research Group, University of Tromsø, Tromsø NO9037, Norway; 3 Department of Neurology, University Hospital of North-Norway, Tromsø NO9038, Norway

**Keywords:** Charcot-Marie-Tooth, Genetic and inherited disorders, Neuromuscular diseases, Mutation analysis, Clinical neurophysiology, Guidelines

## Abstract

**Background:**

Current genetic test algorithms for Charcot Marie Tooth (CMT) disease are based on family details and comprehensive clinical and neurophysiological data gathered under ideal conditions for clinical assessment. However, in a diagnostic laboratory setting relying on external test requisitions and patient samples, such conditions are not always met. Our objective was therefore to perform a retrospective evaluation of the data given in laboratory request forms and to assess their quality and applicability with regard to the recommended algorithms for CMT diagnostics. As we are the main test centre for CMT in Norway our results also provide an overview of the spectrum of gene defects in the Norwegian CMT population.

**Methods:**

Genetic testing was performed according to polyneuropathy type; demyelinating/mixed: *PMP22* duplication, *MPZ*, *EGR2*, *LITAF*, *NEFL*, *PMP22*, *GJB1*, axonal: *MFN2*, *MPZ*, *NEFL*, and *GJB1*.

**Results:**

Diagnostic testing of index patients was requested in 435 of the 549 cases. Seventy-two (16.6%) positive molecular genetic findings were made. The majority (94.6%) of mutation positive cases showed disease onset before 50 years of age. *PMP22* (duplication), *MPZ, GJB1* and *MFN2* mutations constituted 95.8% of the positive findings. Within the nerve conduction study groups, mutation detection rates were; demyelinating 33.8%; mixed 29.0%; axonal 8.8%; unspecified 16.5%.

**Conclusion:**

We suggest a simplified algorithm intended for referral centres, dealing with DNA/blood samples, which involves the assessment of age at onset and neurophysiological data followed by testing of four genes; *PMP22* (duplication), *MPZ*, *GJB1* and *MFN2*. Patients negative for mutations in those four genes should be subjected to evaluation at an interdisciplinary inherited neuropathy clinic with the capacity for extended molecular genetic analysis by next generation sequencing.

## Background

Charcot Marie Tooth disease (CMT) is the most prevalent hereditary neuropathy [1]. In the population of Western-Norway the prevalence has been estimated to 41:100000 [2].

The classic clinical picture of Charcot Marie Tooth disease is characterized by muscular atrophy and weakness in the distal parts of the legs, absence of Achilles tendon reflexes, *pes cavus*, hammertoes and loss of touch and vibratory sensation. Nerve Conduction Velocity (NCV) in the motor median nerve is used to divide autosomal dominant CMT into CMT1 (<38 m/s), CMT2 (>38 m/s) [3] and dominant intermediate CMT (25–45 m/s) [4]. Autosomal recessive CMT is called CMT4 and X-linked CMT CMTX independent of the NCV. Subtypes are defined by the mutant gene and more than 40 CMT associated genetic loci have been identified [5]. The most common CMT subtypes are CMT1A due to a *PMP22* duplication (70–80% of the CMT1 cases) [6,7], CMT2A2 caused by *MFN2* mutations (10–30% of the CMT2 cases) [8,9], CMTX1 (*GJB1*) [4] and CMT4A (GDAP1). Atypical clinical presentations are well documented, particularly for CMT2 [10], CMT4, and CMTX [6]. The recessive CMT4 is associated with an early onset and severe symptoms.

Due to the clinical and genetic heterogeneity, the low sensitivity of genetic testing for CMT2 and scarcity of clinical data, CMT as a group represents a number of challenges for diagnostic laboratories. Recommended algorithms for CMT testing relies on exact clinical details, results from nerve conduction studies (NCS) and inheritance patterns gathered under ideal conditions [11,12] and are therefore suited for specialized neuromuscular or inherited polyneuropathy clinics. Such conditions, however, rarely reflect the reality in everyday practice in laboratories dealing with samples from external patients and, thus, the yield of positive genetic test results in this group is significantly lower [13]. In Norway the vast majority of patients are being tested in this context and, likewise, in a recent UK study [13] almost 2/3rds of investigations were performed on CMT samples from external patients, demonstrating that this is not only a Norwegian phenomenon. In spite of the different test situations and the more heterogeneous nature of the latter group, in depth studies of the diagnostic efficacy of genetic testing of samples sent from external requisitioners are lacking. With regards to guidelines for testing, however, the two groups are often treated as if they were the same. Therefore, our main objective was to assess the quality and applicability of the recommended algorithms for CMT diagnostics in the diagnostic laboratory context. We have reviewed 559 requests for CMT testing received during the period from year 2004 to 2010. We aimed at investigating to which extent available clinical and family information is influencing the success in the identification of disease causing variants. Based on these results we suggest a strategy for molecular genetic CMT testing for diagnostic laboratories which will provide an increase in conclusive test results, a decrease in false positive results and a more efficient use of resources. Furthermore, the results presented here may be of assistance in delineating the patient group that requires the services of an interdisciplinary inherited neuropathy clinic that have the capacity of detailed clinical and neurophysiological studies by experienced clinicians, and also extended molecular genetic analysis by next generation sequencing (NGS).

## Methods

### Patient population

**Figure 1:**
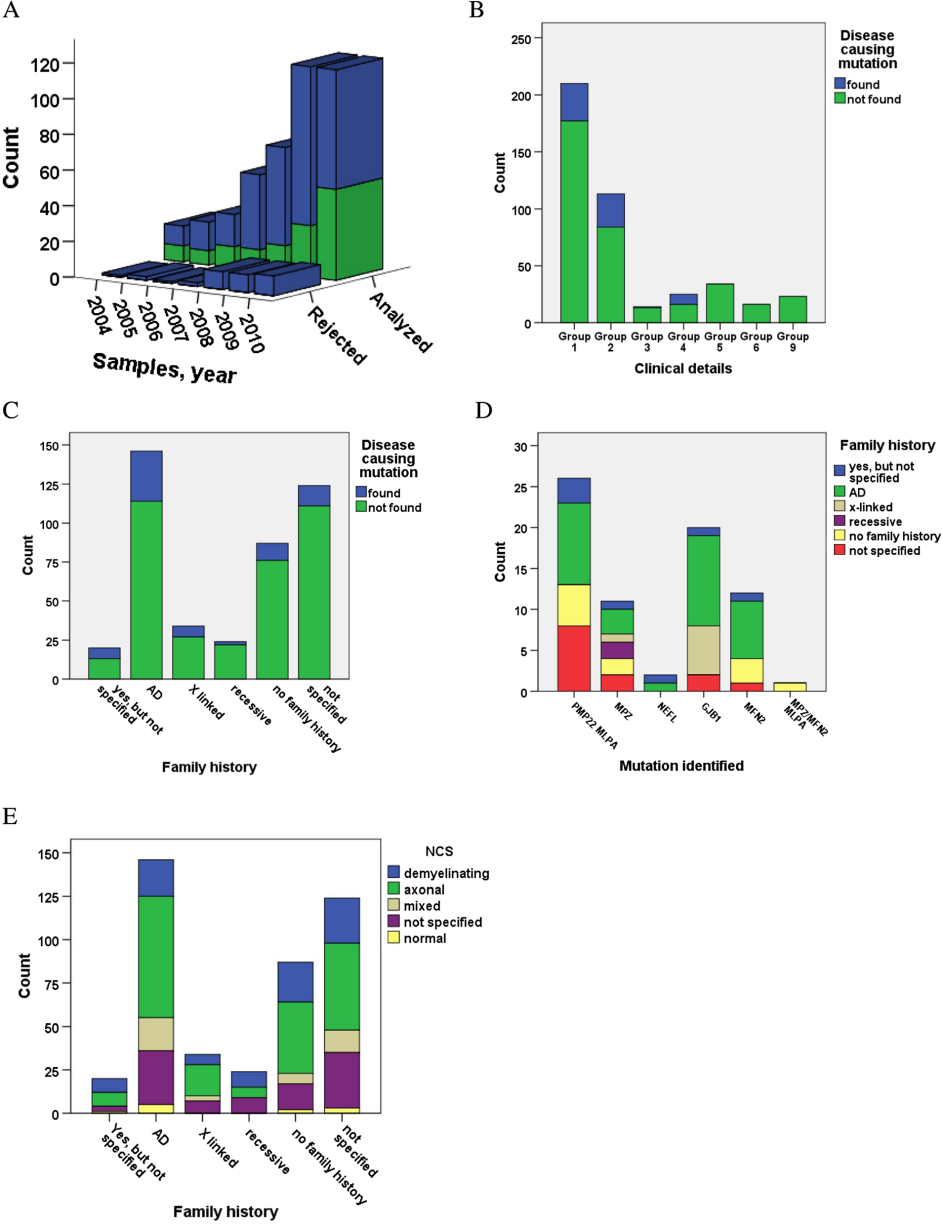
**Recruitment of patient samples, patterns of inheritance, clinical details and genetic findings among the CMT patients. (A)** The annual number of rejected and analyzed samples, 472 in total. Blue color; analyzed in accordance with protocol; green color; analyzed, but with deviation from protocol. **(B)** The sample count and number of findings in the individual clinical groups. 1; Polyneuropathy/CMT? No further information. 2; Specified symptoms of classical CMT. 3; As 2, but specified as severe. 4; As 2, but also with hearing impairment/deafness, pyramidal features, fasciculation, tremor, white matter changes on MRI. 5 Specified symptoms of atypical CMT (Additional file 1: Table S3). 6; Polyneuropathy as part of a more complex clinical picture with additional features usually not seen in association with CMT. 9; Requesting physician primarily suspects alternative diagnosis. **(C)** The sample count and number of findings in relation to mode of inheritance. **(D)** The patterns of inheritance in relation to genetic findings. **(E)** The NCS results in relation to pattern of inheritance.

The Department of Medical Genetics University Hospital of North-Norway is a part of the National Neuromuscular Centre and serves as a referral centre for genetic testing of patients with neuromuscular disorders from all parts of Norway. The annual number of samples received from patients with suspected CMT increased steadily from 25 in 2004 to 147 in 2010; accumulating at a total number of 559 for the seven-year period. Diagnostic, carrier or predictive, testing for known family mutations were requested for 87 patients. These results are not included in this work. Male-to-female ratio in the remaining 472 cases was 1.4:1, and mean age 47.4 (±21.7) years. Based on the information received in the laboratory request forms, 37 requests were rejected. Figure 1A shows the annual sample count and the proportion of rejected samples. The test algorithm was shortened with one or more of the requested analyses in 137/435 investigated index cases. An average of 2.7 genes pro sample were tested in those cases (Additional file 1: Table S1). The rejected group and those with deviation from the test protocol lacked information about nerve conduction studies (NCS) and had sparse or irrelevant clinical information.

### Genetic analyses

DNA was extracted from peripheral blood cells using a Genovision M48 (Qiagen) or Biorobot EZ-1 (Qiagen) systems. Patients with demyelinating polyneuropathy were screened with a CMT1 test battery containing Multiplex Ligation dependent Probe Amplification (MLPA) of the *PMP22* region for the assessment of quantitative alterations and by DNA sequencing of the *MPZ*, *EGR2*, *LITAF*, *NEFL*, *PMP22* and *GJB1* coding exons including at least 20 nt of each flanking intron sequence. Cases with nerve conduction velocities in the intermediate range or with mixed polyneuropathy were also screened with the CMT1 test battery. Patients with axonal polyneuropathy were investigated using a CMT2 test battery which included DNA sequencing of the *MFN2*, *MPZ*, *NEFL* and *GJB1* coding exons including at least 20 nt of each flanking intron sequence. The group of patients displaying normal NCS results was analysed with the CMT2 test panel. Cases, in whom the polyneuropathy type could not be specified, were classified as “deviation from test protocol” if they were not analyzed with both the CMT1 and CMT2 panel. Based on clinical and family information, the *GDAP1* gene was sequenced in individual cases (58/435) representing all polyneuropathy groups. *MPZ/MFN2* MLPA was performed on 229/435 samples.

The primers used for PCR amplification and DNA sequencing are listed in the Additional file 1: Table S2. MLPA reactions were performed using MLPA kits (MRC Holland, Amsterdam, The Netherlands), and PCR products were analyzed by fragment analysis using the Applied Biosystems 3130xl Genetic Analyzer. For CMT1A testing we used the SALSA MLPA KIT P033-B2 CMT1 and for the CMT1A/CMT2A2 testing SALSA MLPA P143 *MFN2-MPZ* probemix (further details are available upon request). Genetic variants were evaluated using the Alamut software (Interactive Biosoftware, San Diego, CA, USA) and in depth literature studies. The variants were categorized into five classes in accordance with the recommendations from the IARC Unclassified Genetic Variants Working Group [14]. Positive findings were defined as uncertain, probable or definite cause of the disease. Variants interpreted as non-pathogenic or likely non-pathogenic were defined as negative findings.

### Data collection, statistics and endpoint measures

From the requisitions the following data were collected and systematized: The year the sample was received, the indication for testing (diagnostic, carrier/predictive or testing rejected), the specialty of the requesting physician (neurology, medical genetics, pediatrics, other), the age at onset of symptoms, the age at testing, whether or not supplemental information had been requested, results from motor NCV in the median nerve, whether or not there were deviations from the test algorithm, if CMT1A had been excluded previously at another laboratory, the type of polyneuropathy as indicated by NCS, the pattern of inheritance and a description of the family history, the number of affected relatives reported in addition to the index case, the gender of the index patient and their relatives, whether or not the mutation was found, and if yes, the name of the gene/mutation and the interpretation of its consequences (from 5 to 1, Additional file 1: Table S3).

**Table 1:** Mutation detection rate in the NCS groups

**Reported result on NCS**	**Disease causing mutation**		**Total count**
	**Found**	**Not found**	
Demyelinating	27	66	93
	(24)	(47)	
	29,0%	71,0%	
	(33.8%)	(66.2%)	
Axonal	17	176	193
	8,8%	91,2%	
Mixed	12	29	41
	29,3%	70,7%	
Not specified	16	81	97
	16,5%	83,5%	
Normal	0	11	11
	0%	100,0%	
Total	72	363	435
	16,6%	83,4%	

In 59 cases CMT1A had already been excluded at other laboratories. The results reported in brackets concern the demyelinating group excluding these cases. The NCS-normal group was analyzed with the CMT2 test panel.

To investigate to which extent available clinical information is influencing the success of genetic diagnostics we found it necessary to stratify our patient population in 9 clinical categories as follows; 1: polyneuropathy/CMT - no further information; 2: Symptoms of classical CMT specified; 3: as 2, but more severe; 4: as 2, but with additional hearing impairment, pyramidal features, fasciculations, tremor or white matter changes on MRI; 5: Symptoms of atypical CMT, HSAN or HMN specified; 6: polyneuropathy with additional features not usually seen in association with CMT (typically ptosis, dysmorphic/multi-systemic features); 7: healthy; 8: testing with regards to a family mutation, symptoms not specified; 9: requesting physician primarily suspects alternative diagnosis (CIDP, congenital myopathy, etc.). Further details, for each category, are shown in Additional file 1: Table S3.

Data from the individual categories were analyzed and data from the two patient groups, the mutation positive and the mutation negative, were compared. Statistical Package for the Social Sciences (SPSS) version 20.0 was used for the statistical analyses.

The research protocol was approved by the Regional Committee for Medical and Health Research Ethics for the counties of Nordland, Troms and Finnmark (REK Nord), and by the Norwegian Data Inspectorate. REK Nord specified that informed consent given by subjects/next-of-kin was not necessary for this study. Procedures were in accordance with the 1964 Declaration of Helsinki and its later amendments.

## Results

### Clinical information and mutation detection rate

Among the 435 cases tested, 72 (16.6%) were found to be mutation positive. They all belonged to the clinical groups 1–4 (Figure 1B). The 73 cases belonging to clinical group 5, 6 and 9 were all mutation negative. This difference in detection frequency between groups 1–4 and 5–9 is highly significant (Pearson chi-square 0.000). Clinical group 4 had the highest detection yield with 36% followed by group 2 (25.7%), group 1 (15.7%) and group 3 (7.1%). The patients in the clinical group 4 had *PMP22* duplications (4), point mutations in *MPZ* (2), *NEFL* (1), *GJB1* (1) and a deletion of exon 13–17 in *MFN2*.

### Patterns of inheritance

Inheritance patterns reported in the 435 cases were autosomal dominant (33.6%), family history not specified (28.5%), single case (20.0%), X-linked (7.8%), autosomal recessive (5.5%) and positive, but with unspecified family history (4.6%). Among the 72 cases with a positive genetic test result, information on family history was reported in 66.6%. In the 363 cases with a negative test result, 48.5% had a positive family history (Pearson chi square p = 0.005). Positive test results were made in all groups with the highest yield in the group with positive, but unspecified family history (35.0%), followed by the autosomal dominant (21.9%), X-linked (20.6%), single case (12.6%), family history not specified (10.5%) and autosomal recessive group (8.3%) (Figure 1C). Seven of the patients with a positive test result had a suspected X-linked inheritance and 6 of these also had a mutation in the *GJB1* gene (Figure 1D). Among 32 patients from families with a suspected dominant inheritance, but with no documented male to male transmission, 11 tested positive on mutations in the *GJB1* gene. The two positive findings in the recessive group were “dominant mutations” in the *MPZ* gene, but no “recessive mutations” were identified. Among 26 cases with *PMP22* duplication, only 10 had been assigned as dominant inheritance. In comparison, 55.0% (11) of the patients with a *GJB1* mutation had been suspected to come from families where CMT segregated as a dominant trait. In 231/435 samples information about the inheritance could not be used to specify CMT subtype because they were either single cases or positive, but with unspecified or unavailable family history. In addition, 47 cases with suspected autosomal dominant, X-linked and recessive inheritance, lacked results of Nerve Conduction Studies (NCS) (Figure 1E).

### Description of the family history

The number of affected relatives was reported for 199 cases. Among 43 patients with a positive genetic test result the mean number of relatives specified was 2.14 ± 1.28. Among 156 patients with a negative genetic test result the mean number of relatives specified was 1.78 ± 0.97. Among the same 199 cases the gender of index and relatives were male and female in 119 cases (59.8%), male only in 53 (26.6%) and female only in 27 cases (13.6%).

### Nerve conduction studies

Polyneuropathy was reported as demyelinating in 21.4%, axonal in 44.4%, mixed in 9.4%, not specified in 22.3%, and normal in 2.5%. The demyelinating and mixed polyneuropathy groups were analyzed with the same testing panel and showed equivalent mutation detection rates (33.8%; 29.3%), (Table 1). Patients, for whom NCS results suggested axonal neuropathy, constituted the largest group and showed the lowest detection rate (8.8%). For patients with NCS-unspecified we obtained a detection rate of 16.5%, similar to the rate found in the material as a whole (16.6%). CMT1A had already been excluded at other laboratories in 59 cases. The mutation detection rate among these 59 cases was 22.0% (3 *MPZ*, 1 *NEFL*, 7 *GJB1* and 2 *MFN2* mutations). The distribution of mutant genes within the NCS groups is similar to what has been reported in the literature. Independent of gender, patients with *GJB1* mutations are represented in all polyneuropathy groups with the majority, however, in the mixed category.

**Figure 2:**
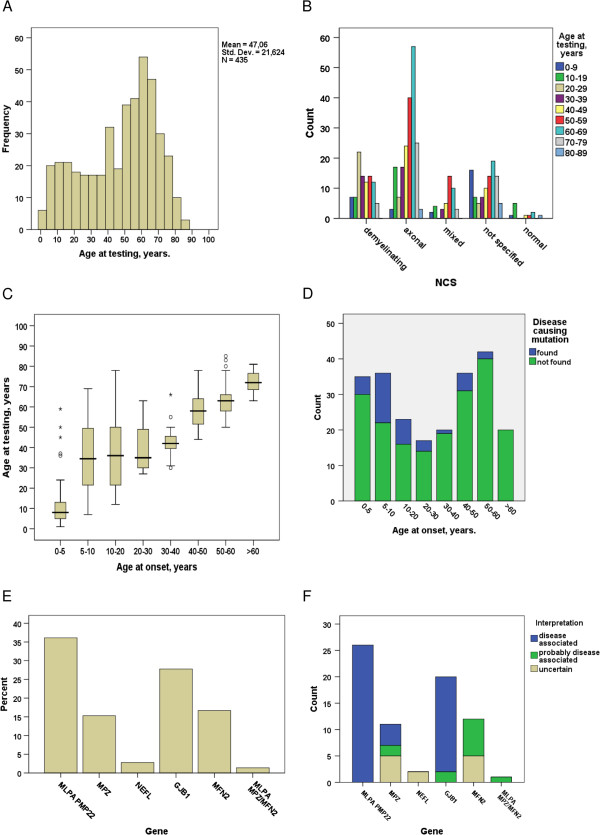
**Clinical, NCS and genetic findings among the CMT patients. (A)** Histogram; the age at testing in 435 patients. **(B)** The distribution of age at testing within the NCS groups. **(C)** Boxplot: the relationship between age at onset and age at testing in 229 samples. **(D)** The distribution of age at disease onset (229 cases) in the group with positive (blue) and negative (green) findings. **(E)** The relative frequency of genetic findings. **(F)** The distribution of genetic findings and interpretation of disease association for 72 mutations.

**Table 2:** **Novel sequence variants not reported in the HGMDp database**^**(†)**^

***Gene***	***cDNA***	***Protein***	***Type***	***het/homo***^***$***^	***Classification***^***£***^
***MPZ***	c.679 A > T	p.Arg227*	Nonsense	het	4
	c.368 G > T	p.Gly123Val	Missense	het	3
	c.410 G > A	p.Gly137Asp	Missense	het	3
***NEFL***	c.1027_1029del	p.Asp343del	In-frame deletion	het	3
***GJB1***	c.775del	p.Leu259*	Deletion	hemi	4
***MFN2***	c.2146_2148dup	p.Ala716dup	In-frame duplication	het	3
	c.250 A > G	p.Lys84Glu	Missense	het	3
	c.612 T > A	p.Asp204Glu	Missense	het	3
	c.653 T > C	p.Leu218Pro	Missense	het	4
	c.692 C > T	p.Ser231Phe	Missense	het	3
	c.1921 T > C	p.Tyr641His	Missense	het	3

^†^HGMDp version 2013.2.

^$^het = heterozygous; homo = homozygous; hemi = hemizygous.

^£^Classification of genetic variants in accordance with the recommendations from the IARC Unclassified Genetic Variants Working Group [14]; 4 = probably disease causing, 3 = variant of uncertain significance.

### Age at disease onset and age at testing

Figure 2A shows the bimodal distribution of age at testing in the group of 435 patients with a second peak starting at 40 years. The age distribution among the NCS groups at testing shows an early peak in the demyelinating group and a late peak in the axonal group, but also in the unspecified group (Figure 2B). Among the 72 cases with a positive genetic test result, the mean age at testing was 37.6 ± 19.4 years, and among the cases with a negative test result it was 48.9 ± 21.6 years. Almost half of the samples received (231/435) were from patients older than 50 years and 23 positive test results were made in this group. Although the differences in the detection yield obtained in the groups younger versus older than 50 years at testing is significant (Pearson chi-square 0.000), the association between age at onset and age at testing is loose (Figure 2C).

Age of onset was reported for 229/435 cases (52.6%). Among these, 37 (16.6%) were found to be positive on genetic testing. Figure 2D compares the age at disease onset in the groups with a positive and negative test result. Most positive findings were made in patients with onset age < 30 years (78.3%) and the highest frequency was associated with onset between 5–20 years (56.7%). The remaining fraction (21.7%) of the positive findings was made in patients with reported onset between 30 and 60 years. However, in the patient material as a whole, 94.6% of the mutation positive patients showed disease onset before 50 years of age. Two mutations were found in the group with onset between 50 and 60 years: a duplication of the *PMP22* region and a missense mutation of uncertain significance (p.Asp35Asn) in the *MPZ* gene.

### Medical specialties and requisitioning

Only index cases were included in this work. All 472 molecular genetic tests were requested by medical doctors. The vast majority was requested from neurology departments (77.1%), but also from departments of medical genetics (7.4%) and pediatrics (10.8%). The remaining 4.7% of the test requests came from medical doctors belonging to other fields of medicine, mostly primary care. Medical genetic departments and “other” often cooperated with a neurologist or pediatrician when they requested testing. Due to sparse clinical information, supplementary information was asked for in 157 cases prior to the selection of testing strategy. The response rate was 61.8% (97/157). The additional information received was not always relevant, particularly when the questions were concerning NCS.

### Molecular genetic findings

Figure 2E shows the relative proportion of positive test results for the various genes tested. The genetic variants detected were interpreted as definitely disease causing in 48, likely disease causing in 12 and uncertain in 12 cases (Figure 2F). Novel mutations not listed in The Human Gene Mutation Database Professional (HGMDp) (http://www.biobase-international.com) version 2013.2, are listed in Table 2. Genetic variants interpreted as likely not, or definitely not disease causing are not dealt with in the present article. The majority (95.8%, 69/72) of the positive molecular genetic findings were either duplication of the *PMP22* region or sequence variants in either one of the *MPZ*, *GJB1* or *MFN2* genes. Testing with *MPZ*/*MFN2* MLPA was performed on 229 samples from all NCS groups and there was one positive finding (deletion of the *MFN2* gene) in a patient with axonal polyneuropathy while the remaining two findings were sequence variants of uncertain significance in the *NEFL* gene. Among the 116 cases analysed with regard to *LITAF*/*SIMPLE* and *EGR2* mutations, and among the 196 cases sequenced with regards to *PMP22* point mutations, no disease causing mutations were found.

## Discussion

All positive genetic test results were made on samples belonging to the clinical groups 1–4, displaying the classical symptoms of CMT. Almost half of these were detected in clinical group 1 in which there were sparse, but relevant, clinical information. No disease causing mutations were found in patients for whom the clinical information gave reason to suspect atypical CMT2, or alternative diagnoses like hereditary sensory and autonomic neuropathy (HSAN), hereditary motor neuronopathy (HMN) or polyneuropathy as part of a more complex clinical picture. The low detection yield in clinical group 3 and in those with a recessive family history, as well as the absence of *GDAP1* mutations in 58 selected patients, imply that we still know little about the genetics of severe/recessive CMT in Norway. None of the patients with a *MFN2* mutation had optic atrophy nor were they categorized as very severely affected.

We find that although a positive family history is associated with an increased probability of positive test results, the apparent pattern of inheritance is of limited value in the selection of testing strategy in most cases. The exceptions are cases with documented male-to-male transmission, leaving *GJB1* testing redundant, as well as for cases where family history suggests a recessive inheritance, indicating that a causative mutation(s) is likely to be found outside the four common genes. However, the family history and subsequent segregation studies are very useful in evaluating genetic variants of unknown or doubtful significance.

Notably, in this study 63.9% of the cases could not be classified in accordance with CMT subtype definitions as they were single cases, cases with positive but unspecified family history, cases where family history was not mentioned, or more rarely because they lacked suitable NCS results.

The reported frequency of *PMP22* duplication in index patients with suspected CMT1 ranges between 23.3% and 60.7% (average 41.8%) across different studies, and in different populations (Table 3) [8,9,15-23]. The reported frequency of *MPZ* mutations ranges between 2.4% and 13.0% (average 5.0%) and *GJB1* mutations between 5.5% and 25.8% (average 8.8%) (Table 3). The mutation detection yields reported in different studies are not directly comparable. This is due to differences in sampling methods, testing strategies and ways of reporting the results. Also, most reports only give a detailed description of patients with positive test results, not the entire cohort. The frequency of *PMP22* duplications among the demyelinating polyneuropathy patients in this material was lower than average (18.7%), whereas the frequency of *MPZ* mutations (6.0%) and *GJB1* mutations (6.7%) were close to average. This may indicate that *PMP22* duplications are less frequent or that a large part of the families with *PMP22* duplication in Norway already were identified by other laboratories before the start of our observation period. Also, a prior exclusion of CMT1A might have been underreported in the patient request forms. A previous study, however, estimated a similar frequency of the *PMP22* duplication (13.6%) in families with CMT in a population in Southern Norway (inhabitants of Akershus County) [24]. The rate of positive molecular diagnostic findings in CMT patients attending neuropathy clinics, compared to external samples sent for molecular genetic investigation, are significantly different. The rate of positive findings in the neuropathy clinic group [12,13] are similar to the high detection rates documented in the literature [6,7,9], whereas the frequencies found in diagnostic laboratories are lower as shown in this and other studies [13], Table 3].

**Table 3:** Mutation detection rates associated with suspected HMSN in various populations

***Population***	***N total***	***Demyelinating/mixed polyneuropathy (CMT1, DSS***^**†**^***, CHN***^**$**^***)***								***Axonal polyneuropathy (CMT2)***				
		**N**	**(%)**							**N**	**(%)**			
			***PMP22*(dup)**	***MPZ***	***EGR2***	***LITAF***	***NEFL***	***PMP22***	***GJB1***		***MFN2***	***NEFL***	***MPZ***	***GJB1***
Spanish [15]	47	35	excl	8.6	-	-	-	2.9	17.1	7	-	-	0	57.1
Korean [16]	57	32	46,9	3.1	3.1	-	3.1	3.1	6.2	18	-	5.6	11.1	5.6
Italian [17]	172	170	57.6	2.4	0	-	-	1.2	7.1	0	-	-	-	-
Australian [18]	224	224^£^	60.7	3.1	-	-	-	1.3	12.1	0	-	-	-	-
Finnish [19]	58	23	excl	13.0	-	-	-	4.3	13.0	29	-	-	0	24.1
Japanese [20]	354	227	23.3	8.8	0.4	0	3.5	4.4	8.4	127	11.3	0	3.9	4.7
British [21]	775	443	28.2	4.2	3.3	-	9.5	5.3	25.8	NR	13.6	-	4.6	2.7
American [6]	153	145	51.6	3.4	0.7	-	0	3.4	5.5^*^	7	-	14.3	0	42.9
Russian [22]	174	108	53.7	4.6	-	-	-	1.9	7.4	32	-	-	0	3.1
European [9]	323	26	-	-	-	-	-	-	-	249	11.2	-	-	-
American [8]	13	0	-	-	-	-	-	-	-	13	23.1	-	-	-
American [23]	39	1	-	-	-	-	-	-	-	38	17,9	-	-	-
**Average**	2389	1407	41.8	5.0	0.5	0	2.2	2.6	8.8	520	12.2	1.3	3.2	10.0
**This study**	435	134	18.7^‡^	6.0	0	0	0.7	0	6.7	193	5.7	0.5	1.0	1.5

The analyses were performed at referral centres for external patients (prospective testing of index cases). The discrepancy between N total and N CMT1/CMT2 are due to additional categories in the studies not listed in the table. - Data not available.

^†^Dejerine-Sottas syndrome.

^$^Congenital hypomyelinating neuropathy.

^£^NCV <50 m/s.

^*^“CMTX”.

^‡^20/107, in 27 cases CMT1A were excluded at another laboratory.

Interestingly, in patients for whom NCS were lacking we obtained a mutation detection rate of 16.5%, similar to the detection rate found in the patient material as a whole (16.6%). This demonstrates that the lack of NCS results should not be used as an absolute exclusion criterion for genetic testing. However, the laboratory offered a wider panel (for both demyelinating and axonal CMT) in those who did not have NCS results specified, hence spending more resources.

The association between age at onset and age at testing is loose. A strong rise in incoming samples from patients of high age, mainly due to a rise in the requested tests for patients with axonal polyneuropathy, is associated with an increased number of phenocopies with axonal polyneuropathy of different genesis. Moreover, on average 42.9% of the patients > 50 years at testing have a negative or unspecified family history, further contributing to this tendency. Age at onset is therefore one of the most important parameters in the decision algorithm. Unfortunately, age at onset was reported only in 52.6% of the cases presented here.

The majority (95.8%) of the positive molecular genetic findings were either duplication of the *PMP22* region or a sequence variant in either one of the *MPZ*, *GJB1* or *MFN2* genes. The present study strongly indicates that none of the genes that have been reported elsewhere to be involved in rare cases of CMT (*LITAF/SIMPLE*, *EGR2*, *PMP22, NEFL, GDAP1* and *MPZ/MFN2* deletions/duplications) are commonly involved in CMT in the Norwegian population. These results are in accordance with other recent publications on the frequency of genetic subtypes [12,13] stating that >90% of mutations in individuals with a positive genetic test are caused by *PMP22*, *GJB1*, *MFN2* and *MPZ*, and that “recessive mutations” are rare. “Recessive mutations” in *SH3TC2* have been reported as a frequent cause of demyelinating CMT in some populations [25]. *SH3TC2* was not a part of the gene panel used in this study, but in a recent paper the frequency was reported to be 0.3% in a large CMT cohort from the United Kingdom [13].

## Conclusion

We suggest a pragmatic two-tier approach to genetic testing in CMT; the first tier should be used by referral centres receiving blood or DNA samples, not patients. Genetic tests in this setting should primarily be selected on the basis of nerve conduction studies. Patients showing demyelinating or mixed polyneuropathy/intermediate nerve conduction velocities, and those in whom the polyneuropathy type is uncertain, should be tested with *PMP22* MLPA and DNA sequencing of the *MPZ* and *GJB1* genes. In cases with documented axonal polyneuropathy DNA sequencing of *MFN2*, *MPZ* and *GJB1* is recommended. A similar conclusion was recently made, based on a study of a CMT population from the UK [13]. Unless there is a clear clinical indication of CMT and positive family history, fifty years at age of onset seems an appropriate cut off age for which testing should be rejected. Good clinical documentation is increasing the probability of detecting disease causing mutations. For cases with sparse clinical information, limited genetic testing as suggested above should be carried out provided that the requests come from experienced neurologists, geneticists or paediatricians. Tests should be restricted to cases with classical CMT, with or without additional features as described in the clinical group 4.

The second tier should be based on interdisciplinary investigation of patients at neuromuscular centres. Several excellent algorithms exist for this purpose [11,12]. Powerful tools, like next generation sequencing (NGS), are nowadays implemented in clinical practice and provide possibilities for more efficient genetic diagnostic service to patients with hereditary polyneuropathies [26]. Of note, we did not analyse all genes known to be associated with CMT. A broad scanning of CMT associated genes likely would have increased the mutation detection rate. Patients negative for mutations in *PMP22*, *GJB1* and *MPZ*, and patients with atypical CMT2, HSAN and HMN should be subjected to extended analysis by NGS and prioritization of the genes to be investigated should be based on detailed studies of the phenotype.

## Competing interests

Potential conflicts of interest; none.

## Authors’ contributions

RØ has taken part in all stages of the project including design and conceptualization of the study, analysis and interpretation of the data and also in drafting and revising of the manuscript for intellectual content. TF has taken part in analysis and interpretation of the data and also in revising of the manuscript for intellectual content. HH has taken part in analysis and interpretation of the data and in revising of the manuscript for intellectual content. BN has taken part in analysis and interpretation of the data and in revising of the manuscript for intellectual content. SIM has taken part in all stages of the project including design and conceptualization of the study, analysis and interpretation of the data and also in drafting and revising of the manuscript for intellectual content. ØN has taken part in all stages of the project including design and conceptualization of the study, analysis and interpretation of the data and also in drafting and revising of the manuscript for intellectual content. All authors read and approved the final manuscript.

## Pre-publication history

The pre-publication history for this paper can be accessed here:

http://www.biomedcentral.com/1471-2350/14/94/prepub

## Supplementary Material

Additional file 1: Table S1 Tests performed on the 137 samples deviating from the test algorithm. **Table S2.** Mutation analysis was carried out by sequencing of all coding regions and their exon-intron boundaries using the listed primers. ^†^3’ untranslated region of the gene. **Table S3.** The variables used in the study. ^†^variable not relevant for this paper. Click here for file
