# Supplementary material for: Diagnostic laboratory testing for Charcot Marie Tooth disease (CMT): the spectrum of gene defects in Norwegian patients with CMT and its implications for future genetic test strategies
Source: BMC Med Genet. 2013 Sep 21;14:94. doi: 10.1186/1471-2350-14-94 (PMC3849068; doi:10.1186/1471-2350-14-94)
Supplement: Additional file 1: Table S1 — Tests performed on the 137 samples deviating from the test algorithm. Table S2. Mutation analysis was carried out by sequencing of all coding regions and their exon-intron boundaries using the listed primers. †3’ untranslated region of the gene. Table S3. The variables used in the study. †variable not relevant for this paper. [file 1471-2350-14-94-S1.doc]

**Table S1 Tests** **performed on the 137 samples deviating from the test algorithm**

| *Gene* | *PMP22* | *MPZ* | *LITAF* | *EGR2* | *PMP22* | *NEFL* | *GJB1* | *MFN2* | *GDAP1* |
| --- | --- | --- | --- | --- | --- | --- | --- | --- | --- |
| Method | MLPA | Seq. | Seq. | Seq. | Seq. | Seq. | Seq. | Seq. | Seq. |
| Count | 101 | 65 | 13 | 13 | 53 | 26 | 72 | 22 | 5 |
| Percent of total (137) | 73.7 | 47.4 | 9.5 | 9.5 | 38.7 | 19.0 | 52.6 | 16.1 | 3.6 |
| Average | 2.7 genes per sample | | | | | | | | |

**Table S2 Mutation analysis was carried out by sequencing of all coding regions and their exon-intron boundaries using the listed primers**

| **Gene** | **Reference**  **sequences** | **Exon** | **Forward primer** | **Revers primer** |
| --- | --- | --- | --- | --- |
| *MPZ* | [NM_000530.6](http://www.ncbi.nlm.nih.gov/entrez/viewer.fcgi?val=NM_000530.4) | 1 | GGGCCCTAGGGGATTTTAAG | GTGGGGATTGCTGAGAGACA |
|  |  | 2 | CCCATAGGTGCATCTGATTCC | AGGATTTCCCCCTCCTTAGC |
|  |  | 3 | AGCTTTGACAGCTGTGTTCTCA | TTCCCCCAACCTATCAGTCC |
|  |  | 4 | CCTCAATGCAGGGCATACAC | GGGTTCTCCTTCCCATCTTG |
|  |  | 5 | GTGGGATGGGAACAGTCAAG | CCATCTCGATGACCATCACC |
| *LITAF/*  *SIMPLE* | NM_004862.3 | 1 | CCCAACGAATTCCCAAATG | AGTGACCTGCAAACCAGAGC |
|  |  | 2 | TCATTGTCTGGTAATGGTTTAAGATG | GGCAGAGTCACTTCGGTCAC |
|  |  | 3 | CAGACGATGAACGCATGCTA | CAAGCATGGTGCAGTTGAGA |
|  |  | 4 | AAGGACATGAACATGGTTTTGG | AAGGCAACTGTGGCTTCTCA |
| *EGR2* | NM_000399.3 | 1 | TGCCCATAAATACTTAGAGCAACA | CACCCCCATCCACCACCTC |
|  |  | 2 | TTCCCCACCTTTTGGACTTT | GGGTAGGCCAGAGAGGAAGA |
|  |  | 2 | TCATCCAGCGTCACCTCTG | TGGGTCTGTTGGGGTACTTG |
|  |  | 2 | CCCCTCCACTCACTCCACTC | TTTGTTGTGCAGCTCCAGTG |
| *PMP22* | NM_000304.2 | 2 | CTAGTGCGCGGGACCCTC | GCAGATTGCCAGAAACTTCC |
|  |  | 3 | CCCCTTTTCCTTCACTCCTC | CCAATAAGCGTTTCCAGCTC |
|  |  | 4 | CTTCTGCTTCTGCTGCCTGT | CATTCTGAGGCCACATCCTT |
|  |  | 5 | CTACCCAGCAATTGTCAGCA | CCACCTCCACTGCTTTCTGT |
| *NEFL* | NM_006158.3 | 1 | GCCTCCCGGCGTATAAATAG | CGGATCTCCTGCTCGTACA |
|  |  | 1 | GCAACGACCTCAAGTCCATC | AGTTCGGCGATCTCCTCTTC |
|  |  | 1 | CGAGAAGCGCATCGACAG | CCCTGGTCTCCACTTTCTGG |
|  |  | 2 | ttgggtattttaggcatgaagg | caggactcctaattacagggttaaa |
|  |  | 3 | tcctgcttgcctttgtgttt | tgagcaaggcttcatttgtct |
|  |  | 4 | tttgcaaataaaacctttgcag | CCACCGAAGGTTCAAAGGAC |
| *MFN2 MFN2* | **NM_014874.3** | 1^†^ | ggaactacagcccccatgat | gccgagctgctcaggact |
|  |  | 2^†^ | tgtgcacagtcaggattggt;  cggatccatctcccttttct | cagaggtccaggagagcaca |
|  |  | 3 | tgagacactgttcttatctcaccgtcc | ccacatgatttcaagagcagagca |
|  |  | 4 | cgtttagggtaagcagggccg | gaggaaagcaatgaattctgaagca |
|  |  | 5 | gtcagaggtttgggcctggg | agggtctcccattcacctcca |
|  |  | 6 | gggtgatatccgggaaagaagc | ccctgggacatctgcagggta |
|  |  | 7/8 | tgagggccaggcctgatttc | cccagtggcaggcagtgct |
|  |  | 9 | ttccctcactctgtcgccca | tgacagactcctcagcacgagaca |
|  |  | 10/11 | gccagtggcttggtttctgg | gccctcctcaccaggctgtc |
|  |  | 12 | tttgtgcccaccacctgacc | aatggaacccaaacggcagc |
|  |  | 13/14 | gagctgaggaggctgctggtt | ccgcatctgatctttgggca |
|  |  | 15 | atgctcagtctcacgggcca | tcactgcttagaacaaatccaggtca |
|  |  | 16 | gggtcaggatggctcctgct | gatcccaccgaggccagaag |
|  |  | 17 | gcctcggtgggatcaaagga | aaagctggccctgcacacct |
|  |  | 18 | ggccgtggtgatgctgagtg | catggtacacatctaacacgaatgagc |
|  |  | 19 | tgtggccttgggaagttatt | CTGTCCATAGCTGTCGCTGA |
| *GJB1* | NM_000166.5 | 2 | CCAGCTTTCTGACAGCTTGCT | GCATAGCCAGGGTAGAGCAG |
|  |  | 2 | CCTACACCTGGAGGAGGTGA | TAGCCAGGGAAGGAAGGTTT |
| *GDAP1* | NM_018972.2 | 1 | ACCTCCCAGGTGCACTCC | AGGACACTGGAGGCGGATT |
|  |  | 2 | TGTTCTTTCCTTACTTGTTAATTCCAG | TTTGTGTGCTTATGGAAATGGA |
|  |  | 3 | GCATCAGGCCATTTCAAACT | CCAATTGAGCAAGTGAAGCA |
|  |  | 4 | TAGACAGGGTAAGCCCAAGG | CCAGCATGCTCAATATCTGC |
|  |  | 5 | TCTCGTTGTCTAAAATAGGCTGAA | CAGAACGTTTTTGGTGAGACC |
|  |  | 6 | CCTTAAGGGTGAGACCACTGA | ACTCAATAAGACAGACTCAGAGAGC |

**Table S3 The variables used in the study**

| First CMT test performed, year: Index case or relative tested for family mutation  Indication for testing: Diagnostic, carrier testing/predictive testing^†^, or testing was rejected by the laboratory^†^  Specialty of requiring physician: Neurology, medical genetics, pediatrics, or other  Supplemental information: Asked for/received  Age (years): At onset of symptoms and at the time of testing  Family history - assessment of inheritance pattern: Positive, but not specific, autosomal dominant, x-linked, recessive, single case, family history not mentioned  Description of the family history: Single case, two or more affected siblings, affected over two or more generations with “male to male” transmission, affected over two or more generations without “male to male” transmission  Number of affected relatives in addition to the index case  Gender of index patient and relatives | Motor NCV in the median nerve reported  Polyneuropathy type indicated by NCS studies: Demyelinating, axonal, mixed, normal, not specified  Clinical information: **1**: Polyneuropathy/CMT, no further information. **2**: Specified symptoms of classical CMT. **3**: As 2, but severe. **4**: As 2, with additional features known to be associated with CMT. **5**: Specified symptoms of atypical CMT: Pure involvement of the sensory or autonomous nervous system; Pure motor nerve involvement; Symptoms of upper limbs > lower limbs; Vocal cord paresis; Proximal involvement > distal involvement. **6**: Polyneuropathy combined with additional features not described in association with CMT. **7**: Healthy^†^. **8**: Testing of a family mutation, symptoms not specified^†^. **9**: Alternative diagnosis primarily suspected.  Test algorithm followed: Yes, no, or testing rejected  CMT1A excluded at an other laboratory  Mutation found: Name of the gene mutation and the classification of the mutation  Individual genes tested on each sample |
| --- | --- |
